# Telemedicine in the Management of Type 1 Diabetes

**DOI:** 10.5888/pcd15.170168

**Published:** 2018-01-25

**Authors:** Timothy Xu, Shreya Pujara, Sarah Sutton, Mary Rhee

**Affiliations:** 1 Mayo Clinic School of Medicine, Rochester, Minnesota; 2 Emory University, Atlanta, Georgia; 3 Central Alabama Veterans Health Care System, Montgomery, Alabama; 4 Atlanta Veterans Affairs Medical Center, Decatur, Georgia

## Abstract

**Background:**

Veterans with type 1 diabetes who live in rural Alabama and Georgia face barriers to receiving specialty diabetes care because of a lack of endocrinologists in the Central Alabama Veterans Health Care System. Telemedicine is a promising solution to help increase access to needed health care. We evaluated telemedicine’s effectiveness in delivering endocrinology care from Atlanta-based endocrinologists.

**Methods:**

We conducted a retrospective chart review of patients who were enrolled in the Atlanta VAMC Endocrinology Telehealth Clinic from June 2014 to October 2016. Outcomes of interest were hemoglobin A1c levels, changes in glycemic control, time savings for patients, cost savings for the US Veterans Health Administration, appointment adherence rates, and patient satisfaction with telehealth.

**Results:**

Thirty-two patients with type 1 diabetes received telehealth care and in general received the recommended processes of diabetes care. Patients trended toward a decrease in mean hemoglobin A1c and glucose variability and a nonsignificant increase in hypoglycemic episodes. Patients saved 78 minutes of travel time (one way), and the VA saved $72.94 in travel reimbursements per patient visit. Patients adhered to 88% of scheduled telehealth appointments on average, and 100% of surveyed patients stated they would recommend telehealth to other veterans.

**Conclusions:**

Specialty diabetes care delivered via telemedicine was safe and was associated with time savings, cost savings, high appointment adherence rates, and high patient satisfaction. Our findings support growing evidence that telemedicine is an effective alternative method of health care delivery.

## Introduction

The diabetes epidemic is continuously growing in America and affects 29.1 million Americans (9.3% of the US population) (1). The burgeoning prevalence of diabetes has created an increase in demand for specialty diabetes care. However, there is a nationwide shortage of approximately 1,500 full-time endocrinologists (2), creating a disparity between diabetes care and specialty diabetes providers. 

Patients who live in rural areas, approximately 20% of the US population, have more barriers to receiving specialty care. Barriers such as long travel distances and costly expenses to urban areas where specialty care is often available (3,4) create challenges for these patients to achieve good health (4). Telemedicine, the exchange of medical information via electronic communications such as clinical video telehealth (CVT) (real-time videoconferencing between patients and providers), has emerged as a promising solution (5,6). The US Veterans Health Administration (VHA) created the Telehealth Services Program to increase access to specialty medical care for veterans with limited access (7). In 2014, the Atlanta Veterans Affairs Medical Center (VAMC) Endocrinology Telehealth Clinic was established to deliver specialty diabetes care to patients with type 1 diabetes in the Central Alabama Veterans Health Care System (CAVHCS); because the CAVHCS serves rural communities in Alabama and west Georgia, specialty diabetes care is often inaccessible for these patients.

We characterized the effectiveness of the Atlanta VAMC Endocrinology Telehealth Clinic in improving diabetes outcomes for patients with type 1 diabetes and increasing their access to specialty diabetes care. We studied patients with type 1 diabetes because the Atlanta VAMC Endocrinology Telehealth Clinic was created to increase access to specialty care for type 1 diabetes patients who manage their condition with insulin pump therapy. We hypothesized that management of type 1 diabetes via CVT leads to improvements in glycemic control, saves costs for the VHA, saves time for patients, and is associated with high appointment adherence and patient satisfaction.

## Methods

CAVHCS serves more than 134,000 veterans in 43 counties of Alabama and Georgia but does not employ a local endocrinologist. In 2014, the Atlanta VAMC Endocrinology Telehealth Clinic was established to increase access to specialty care for type 1 diabetes for CAVHCS patients. Without telehealth, CAVHCS patients have to travel to the Veterans Affairs (VA) medical centers in either Birmingham, Alabama, or Atlanta, Georgia, to receive in-person specialty care. With telehealth, patients travel to local community-based outpatient clinics for their telehealth appointment, where they check in as they would for a regular face-to-face appointment; they have their vital signs checked, go to a patient care room with a webcam or dedicated telehealth monitor, and have a CVT consultation from an Atlanta-based endocrinologist with in-person assistance from a telehealth pharmacist. Visits typically last 30 to 60 minutes.

We conducted a retrospective chart review of patients with type 1 diabetes who received care through the Atlanta VAMC Endocrinology Telehealth Clinic from June 2014 to October 2016. We collected data about changes in glycemic control, telemedicine’s capacity to save costs for the VHA and time for patients, patient adherence to telemedicine appointments, and patient satisfaction with telemedicine. Data were stored in REDCap, a secure web-based database application. Our use of REDCap was sponsored by the Atlanta Clinical and Translational Science Institute. This study was approved by the Emory institutional review board and the Atlanta VA Research and Development Committee.

To assess diabetes management, we collected data on recommended processes of diabetes care: blood pressure management, eye screening, urine microalbumin-to-creatinine ratio, and lipid panels (triglycerides, high-density lipoprotein cholesterol, low-density lipoprotein cholesterol). We also assessed whether patients received drug prescriptions for which they were eligible, specifically statins and aspirin.

To assess diabetes outcomes, we collected data on change in glycemic control, specifically hemoglobin A1c levels, 2-week frequency and severity of hypoglycemia, 2-week frequency and severity of hyperglycemia, and plasma glucose variability. Hemoglobin A1c indicates average plasma glucose concentration over 2 to 3 months and predicts diabetes complications (8,9). Hypoglycemia is defined as low plasma glucose concentration, and severe hypoglycemia may lead to unconsciousness (9). We defined hypoglycemia as a plasma glucose level of less than 70 mg/dL and severe hypoglycemia as less than 40 mg/dL. Hyperglycemia is defined as high plasma glucose concentration, which may lead to long-term complications such as diabetic retinopathy, nephropathy, and neuropathy (10). We defined hyperglycemia as a plasma glucose level of more than 250 mg/dL and severe hyperglycemia as more than 300 mg/dL. We reviewed patients’ insulin pump downloads or patients’ glucose logs over a 2-week period to determine frequency of hypoglycemia and hyperglycemia. Lastly, average glucose variability was defined as the standard deviation (SD) of all plasma glucose levels in the 2-week period. Data on glycemic control were collected at baseline visits, 6 month follow-up visits (±1 month), and 12 month follow-up visits (±1 month).

Cost savings for the VHA were calculated on the basis of the difference between patient travel reimbursement costs associated with in-person visits at VA medical centers in either Birmingham, Alabama, or Atlanta, Georgia, and costs associated with telemedicine visits at community-based outpatient clinics. Travel reimbursements were calculated using reimbursement rates published by the VHA’s Beneficiary Travel Benefits program, which was 41.5 cents per mile with a $6 patient deductible (11). Patients who traveled more than 75 miles one way were eligible for VA-reimbursed overnight lodging, and lodging costs of $75 were added to the travel cost for an in-person visit. Time savings for patients were calculated using Google Maps (Google Inc) and were based on the difference in estimated time to travel to community-based outpatient clinics versus the nearest VA medical center in either Atlanta, Georgia, or Birmingham, Alabama.

To evaluate telemedicine appointment adherence, we recorded the number of CVT appointments missed (patient did not show up), cancelled, and scheduled. Telemedicine appointment adherence was reported as the ratio of the number of CVT appointments in which the patient showed up to the number of CVT appointments scheduled, excluding the number of appointments cancelled by the patient in advance. To assess patient satisfaction with telemedicine, we administered via telephone a satisfaction survey published by the VA Telehealth Services Program. Patients were surveyed about telemedicine’s usability and convenience, and their satisfaction was measured using a Likert Scale with scores ranging from 1 through 5 (1 = “strongly agree” and 5 = “strongly disagree”).

Data analysis was performed using Microsoft Office Excel 2010 (Microsoft Corporation), SPSS version 23.0 (IBM Corp), and SAS version 9.4 (SAS Institute Inc). To analyze changes in diabetes outcomes, we conducted paired *t* tests from baseline data, 6-month follow-up data, and 12-month follow-up data. Significance was set at *P* < .05. To analyze patient satisfaction survey results, we calculated the median, mean, and SDs of patient responses to each survey question.

## Results

### Demographic characteristics

Among 54 patients enrolled in the Atlanta VAMC Endocrinology Telehealth Clinic, 32 patients had type 1 diabetes (Figure). Of the 32 patients with type 1 diabetes, 17 had follow-up visits at 6 months, and 9 had follow-up visits at 12 months. Telehealth patients with type 1 diabetes were predominately male (n = 29, 91%) and white (n = 27, 84%) (Table 1). Mean age was 53.5 years and mean body mass index was 27.6 kg/m^2^. Comorbidities and diabetes complications were highly prevalent at baseline in this patient population; most patients had hyperlipidemia (n = 26, 81%) and diabetic neuropathy (n = 23, 72%).

**Figure:**
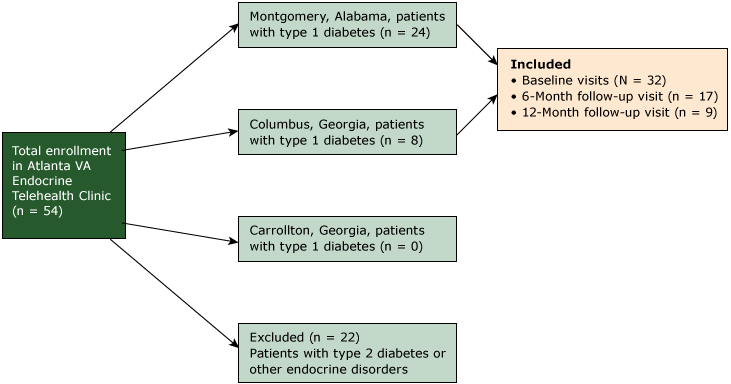
Diagram showing criteria for inclusion in a study of patients (N = 32) enrolled in the Atlanta VA Telehealth Endocrine Clinic, June 2014 to October 2016. Abbreviation: VAMC, Veterans Affairs Medical Center.

**Table 1:** Demographic Characteristics of Patients, Study of Patients (N = 32) Enrolled in the Atlanta VA Telehealth Endocrine Clinic, June 2014 to October 2016

Characteristic	Telehealth Patients With Type 1 Diabetes at Baseline (N = 32)^a^
**Mean (SD) age, y**	53.5
**Sex**	
Male	90.6
Female	9.4
**Race**	
White	84.4
Black	15.6
**Primary care location**	
Montgomery, Alabama	75.0
Columbus, Georgia	25.0
Carrollton, Georgia	0
**Mean (SD) body mass index, kg/m^2^**	27.6
**Mean (SD) duration of diabetes, y**	24.7
**Insulin pump use**	75.0
**Continuous glucose monitor use**	18.8
**Hypertension**	46.9
**Hyperlipidemia**	81.3
**Hypothyroidism**	28.1
**Tobacco use**	21.9
**Microvascular diseases **	
Neuropathy	71.9
Nephropathy	21.0
Retinopathy	40.6
**Macrovascular diseases**	
Coronary Artery disease	25.0
Cerebrovascular disease	12.5
Peripheral vascular disease	3.1

a Values are percentages unless otherwise indicated.

Telehealth patients generally received the standard processes of diabetes care (Table 2) (12). At baseline, 94% patients (30 of 32) had a diabetic retinopathy eye screening within the preceding 2 years, and 100% (9 of 9) received the recommended eye screening at 12-month follow-up. Furthermore, 81% of patients (26 of 32) had their urine microalbumin-to-creatinine ratio measured at baseline, which increased to 89% (8 of 9) at 12-month follow-up. Of patients who were eligible for statin use, 89% (24 of 27) were prescribed a statin, and 64% patients who were eligible for aspirin use (14 of 22) were prescribed aspirin. At 12-month follow-up, 88% of eligible patients (7 of 8) were prescribed a statin, and 50% of eligible patients (1 of 2) were prescribed aspirin. When seen at baseline visits and at 6-month and 12-month follow-up visits, all patients had received the recommended blood pressure measurements and lipid panels.

**Table 2:** Maintenance of Standard Processes of Diabetes Care, Study of Patients (N = 32) Enrolled in the Atlanta VA Telehealth Endocrine Clinic, June 2014 to October 2016

American Diabetes Association 2016 Guideline	Monitoring	Percentage^a^ of Patients With Recommended Care at Baseline	Percentage^a^ of Patients With Recommended Care at 6 Months	Percentage^a^ of Patients With Recommended Care at 12 Months
Blood pressure	Every routine visit	100 (32 of 32)	100 (17 of 17)	100 (9 of 9)
Diabetic retinopathy eye exam	Every 1 year	93.7 (30 of 32)	94.1 (16 of 17)	100 (9 of 9)
Urine microalbumin-to-creatinine ratio	Every 1 year	81.3 (26 of 32)	88.2 (15 of 17)	88.9 (8 of 9)
Lipid panel (triglyceride, HDL, and LDL levels)	Every 1 year	100 (32 of 32)	100 (17 of 17)	100 (9 of 9)
Statin use	Eligibility: aged >40 y or history of CVD	88.9 (24 of 27)	100 (15 of 15)	87.5 (7 of 8)
Aspirin use	Eligibility: aged >50 or history of CVD	63.6 (14 of 22)	69.2 (9 of 13)	50.0 (1 of 2)

Abbreviations: CVD, cardiovascular disease; HDL, high-density lipoprotein cholesterol; LDL, low-density lipoprotein cholesterol.

a Values in parentheses are number of patients who adhered to recommendation out of total number.

### Diabetes outcomes and glycemic control

Mean hemoglobin A1c levels decreased overall from baseline (8.7%) to 6-month (8.2%) and 12-month (8.1%) follow-up, although the change was not significant. After 6 months and 12 months, patients also had a mean increase in average frequency of hypoglycemia per 2 weeks of blood glucose levels less than 70 mg/dL and less than 40 mg/dL, although these trends were not significant. The mean frequency of hypoglycemia of glucose less than 70 mg/dL was 3.3 hypoglycemic episodes per 2 weeks at baseline, 3.3 at 6-month follow-up, and 6.2 at 12-month follow-up. The average frequency of hypoglycemic episodes per 2 weeks of glucose less than 40 mg/dL was 0.2 at baseline, 0.2 at 6-month follow-up, and 0.6 at 12-month follow-up. Clinically, the difference in severe hypoglycemia (<40 mg/dL) was insignificant, but hypoglycemia of glucose less than 70 mg/dL increased overall.

The average frequency of hyperglycemia every 2 weeks increased from baseline to 6-month follow-up but was stable after 12 months. This trend was observed in hyperglycemic episodes of glucose greater than 250 mg/dL and greater than 300 mg/dL but was not significant. The mean frequency of hyperglycemia greater than 250 mg/dL was 16.3 at baseline, 22.5 at 6-month follow-up, and 16.2 at 12-month follow-up. For hyperglycemic episodes greater than 300 mg/dL, the mean frequency was 4.0 at baseline, 5.4 at 6-month follow-up, and 3.8 at 12-month follow-up.

Lastly, there was a nonsignificant trend toward a decrease in mean 2-week blood glucose levels at 6-month and 12-month follow-up. Mean daily blood glucose level was 79.2 mg/dL (SD, 20.4 mg/dL; n = 27) at baseline, 76.2 mg/dL (SD, 15.7 mg/dL; n = 16) at 6 months, and 76.4 mg/dL (SD, 19.7 mg/dL; n = 9) at 12 months.

### Time and cost savings

Patients saved a median of 78 minutes of one-way traveling time, and the VHA saved a median of $72.94 per patient visit in travel reimbursement. If Atlanta VAMC Endocrinology Telehealth patients received follow-up appointments every 3 months as recommended, each patient would save 624 minutes of traveling time per year, which corresponds with VHA savings of $9,336.32 per year in reimbursements to the 32 patients with type 1 diabetes.

### Telehealth appointment adherence and patient satisfaction with telemedicine

Telehealth patients had a median of 5 scheduled appointments (range, 1–10 scheduled appointments). Patients were adherent to their telehealth appointments; at least half of the patients attended 100% of their appointments, and mean adherence rate was 87.8% (SD, 17.8%; range, 50.0%–100%).

Twenty-two (69%) telehealth patients with type 1 diabetes completed the survey about their satisfaction with telehealth care. Patients perceived the endocrinology care they received during their telemedicine appointments favorably; 100% of respondents agreed or strongly agreed that they were satisfied with telehealth (Table 3). Furthermore, 90.9% respondents strongly agreed with the statement that they would recommend telehealth to other veterans, and 90.9% respondents agreed or strongly agreed that they would rather use telehealth than travel long distances to see their providers. Two patients who preferred in-person care over telehealth stated that seeing their physician face-to-face was important to them.

**Table 3:** Patient Responses to Telehealth Satisfaction^a^ Survey, Study of Patients With Type 1 Diabetes (N = 32) Enrolled in the Atlanta VA Telehealth Endocrine Clinic, June 2014 to October 2016

Telehealth Patient Satisfaction Survey Question	Median	Mean (SD)
I felt comfortable with the equipment used.	5.00	4.91 (0.29)
I was able to see the clinician clearly.	5.00	4.95 (0.21)
I was able to hear the clinician clearly.	5.00	5.00 (0)
There was enough technical assistance for my meeting with the clinician.	5.00	4.95 (0.21)
My relationship with the clinician was the same during this session as it is in person.	5.00	4.18 (1.01)
The location of the telehealth clinic is convenient for me.	5.00	4.68 (0.65)
My needs were met during the session.	5.00	4.95 (0.21)
I received good care during the session.	5.00	4.95 (0.21)
The telehealth clinic provided the care I expected.	5.00	4.95 (0.21)
Overall, I am satisfied with the telehealth session.	5.00	4.91 (0.29)
I would recommend this type of session to other veterans.	5.00	4.77 (0.75)
I would rather use telehealth to receive this service than travel long distance to see my provider.	5.00	4.59 (1.05)

a Patient satisfaction was measured using a Likert Scale (from 1 through 5), where 1 indicated “strongly agree” and 5 indicated “strongly disagree.”

## Discussion

Our findings suggest that telemedicine is a safe method of delivering type 1 diabetes care to rural patients. Telehealth patients in our study experienced improvements overall in diabetes outcomes, although our findings were not significant. Patients also had an increased mean frequency of hypoglycemia. Our observation of increased hypoglycemic episodes is consistent with literature that suggests improved glycemic control, indicated by lower hemoglobin A1c levels, is correlated with an increased frequency of hypoglycemia (13).

Our findings are in line with those of other studies that suggest that diabetes care via telemedicine is comparable to in-person diabetes care. For example, in a recent randomized controlled trial of 282 diabetes patients, those who received telemedicine consultation had a −1.01% decrease in hemoglobin A1c compared with a −0.68% decrease in hemoglobin A1c in those receiving in-person consultation, although the change was nonsignificant (14). Our findings, which demonstrated a 0.6% decrease in hemoglobin A1c at 12 months of telemedicine follow-up consultation, complement this study’s findings and growing evidence that suggests that telemedicine is a viable alternative for in-person care.

Previous studies also demonstrated telemedicine’s effectiveness in delivering diabetes care to rural patients. Wood et al described telemedicine’s use in pediatric type 1 diabetes care for patients in rural Wyoming, demonstrated equivalency between telemedicine and in-person visits, and found that patients received more follow-up visits after telemedicine’s implementation (15). Similarly, Wagnild et al described the use of telecommunications for diabetes patients in Montana and found that patients showed improvements in hemoglobin A1c levels, blood pressure, and diabetes knowledge (16). Our findings are consistent with literature that suggests that telemedicine may effectively deliver diabetes care to rural patients.

Our study has limitations. First, the referring diabetes specialty provider at CAVHCS also independently manages the diabetes treatment of many of the patients enrolled in the telehealth clinic, in some cases just before referral to the telehealth clinic but mostly with select patients between telehealth visits as needed. Thus, telehealth patients’ glycemic control before baseline visits and afterward may have been better than that of patients who receive care only from primary care providers (17). However, use of midlevel providers such as pharmacists and nurses is common across the VA health system, is an integral part of the VA-established Patient Aligned Care Team model, and may represent the patient-centered care model in use (18).

Another limitation was significant loss of follow-up. Many patients had follow-up visits that did not meet our study criteria of 6- and 12-month follow-up points. This apparent loss of follow-up may have been because the Atlanta VA Telehealth Endocrinology Clinic is available only once per week. As more patients enrolled in the clinic over time, the intervals between follow-up appointments necessarily increased. Therefore, some patients did not have an appointment scheduled at the 6-month point (5–7 months after baseline) or the 12-month point (11–13 months after baseline). Thus, if a patient had an appointment before 11 months or over 13 months after their initial appointment, they would not have been included for the 12-month follow-up analysis. Our follow-up data may have been further confounded by the possibility that patients with worse glycemic control needed more frequent follow-up and thus were more likely to have 12-month follow-up data.

Additionally, our study used convenience sampling of patients enrolled in the Atlanta VAMC Endocrinology Telehealth Clinic. Our findings may not accurately represent patients with type 1 diabetes in the general population because all our patients were veterans seen at the VA and most had insulin pumps, which are associated with better glycemic control compared with insulin injections (19). Furthermore, our evaluation of aspirin use may have been limited by inconsistent documentation of its use, because many patients purchase it over-the-counter at local drug stores, leading to an underestimation of its use.

Lastly, our limitations include self-selection bias and small sample size. Self-selection bias may have affected our satisfaction survey results because patients who prefer telemedicine may be more likely to enroll in telehealth clinics, whereas patients who prefer in-person care may be more likely travel to VA medical centers to receive treatment. Furthermore, our small sample size limited our statistical power and generalizability. However, these limitations were inherent in our study design, because we conducted a retrospective review of only patients enrolled in our telehealth clinic.

One of telemedicine’s most important benefits is its ability to increase access to health care. Distance is a significant factor for many veterans living in remote and rural areas seeking health care, because travel distance is negatively correlated with use of outpatient services (20). The VA has mitigated this issue by providing travel reimbursement and bus services for patients, but telemedicine further promotes health care accessibility for rural patients. Another important aspect of telemedicine is its acceptance by patients and providers. Our study demonstrates that most patients are satisfied with telemedicine care, believe that telemedicine appointments are convenient, and would recommend telemedicine to other veterans. Our findings are consistent with those of studies that report that both patients and providers are highly satisfied with telemedicine (21–24).

Lastly, our findings suggest that telemedicine leads to substantial cost savings and complement findings from studies that demonstrate telemedicine’s cost-saving capacity in larger health care systems. For example, the use of telemedicine in 7 rural hospital emergency departments in Mississippi decreased the hospitals’ expenditures from $7.6 million to $1.1 million during a 5-year period with no apparent effect on clinical outcomes (25). If the VHA implements telemedicine on a broader scale, veterans could receive more accessible patient-centered care, and the VHA could benefit from significant cost savings.

Our findings suggest that telemedicine delivers safe diabetes care to rural veterans and supports growing evidence that suggests that telemedicine is an effective alternative method of health care delivery. Additionally, telemedicine is associated with cost savings for the VHA, time savings for patients, high appointment adherence, and high patient satisfaction. Future studies with larger, more representative samples of patients with type 1 diabetes are needed to elucidate telemedicine’s effectiveness in providing health care to broader patient populations.
